# Detection of Osteoporosis from Percussion Responses Using an Electronic Stethoscope and Machine Learning

**DOI:** 10.3390/bioengineering5040107

**Published:** 2018-12-05

**Authors:** Jamie Scanlan, Francis F. Li, Olga Umnova, Gyorgy Rakoczy, Nóra Lövey, Pascal Scanlan

**Affiliations:** 1 The School of Computing, Science & Engineering, Newton Building, University of Salford, Salford, Greater Manchester M5 4WT, UK; j.scanlan1@edu.salford.ac.uk (J.S.); o.umnova@salford.ac.uk (O.U.); george.rakoczy04@gmail.com (G.R.); 2 Warrington Hospital, Lovely Lane, Warrington, Cheshire WA5 1QG, UK; n.lovey@nhs.net; 3 Taybank Medical Centre, 10 Robertson Street, Dundee, DD4 6EL, UK; pscanlan@nhs.net

**Keywords:** osteoporosis, electronic stethoscope, vibro-acoustics, machine learning, resonant frequency, impulse response, signal processing, pattern recognition, classification

## Abstract

Osteoporosis is an asymptomatic bone condition that affects a large proportion of the elderly population around the world, resulting in increased bone fragility and increased risk of fracture. Previous studies had shown that the vibroacoustic response of bone can indicate the quality of the bone condition. Therefore, the aim of the authors’ project is to develop a new method to exploit this phenomenon to improve detection of osteoporosis in individuals. In this paper a method is described that uses a reflex hammer to exert testing stimuli on a patient’s tibia and an electronic stethoscope to acquire the impulse responses. The signals are processed as mel frequency cepstrum coefficients and passed through an artificial neural network to determine the likelihood of osteoporosis from the tibia’s impulse responses. Following some discussions of the mechanism and procedure, this paper details the signal acquisition using the stethoscope and the subsequent signal processing and the statistical machine learning algorithm. Pilot testing with 12 patients achieved over 80% sensitivity with a false positive rate below 30% and accuracies in the region of 70%. An extended dataset of 110 patients achieved an error rate of 30% with some room for improvement in the algorithm. By using common clinical apparatus and strategic machine learning, this method might be suitable as a large population screening test for the early diagnosis of osteoporosis, thus avoiding secondary complications.

## 1. Introduction

Osteoporosis (OP) is an asymptomatic disease that affects the bone re-modelling process. This results from a period of bone loss which leads to increased skeletal fragility and an increased risk of fracture. Osteoporosis is significantly prevalent in people over the age of 50, and affects post-menopausal women earlier. One in three women and one in five men over the age of 50 will sustain a fracture attributable to osteoporosis, according to the International Osteoporosis Foundation [1]. Globally, osteoporosis affects more than 200 million people worldwide, being the cause of more than 8.9 million fractures annually [2,3]. Healthcare services are heavily burdened by the costs of treating fractures and their complications due to undetected and untreated osteoporosis, and the quality of life of such patients is considerably compromised or detrimentally degraded. Healthcare professionals have shown that early diagnosis and timely treatment is the way of preventing further complications and fractures. However the lack of a simple and practical screening test has been a major concern, with current methods not fit for this purpose. 

The most common diagnostic method to detect OP is using dual energy X-ray absorptiometry (DEXA/DXA) to measure bone mineral density (BMD), although it is arguable if the BMD is an accurate diagnostic metric [4]. The World Health Organisation (WHO) recommended a statistical measure based on DEXA called the “t-score” as a diagnostic criterion [5]. The t-score is a comparison of one’s BMD with that of a population of healthy 30-year-old pre-menopausal women and is reported in standard deviations (SD). A t-score below −2.5 SD is deemed as being osteoporotic. A t-score between −1.0 to −2.5 is labeled as osteopenia, meaning that the BMD is notably below a normal measure but not fully osteoporotic. However, the BMD is not a reliable measure of the bone’s strength and can only imply bone quality [6,7]. DEXA scans are not readily accessible to general practitioners’ (GPs) clinics for screening testing but are only available in general hospitals via primary care professionals’ referrals, and often have long waiting lists [8,9,10].

One can tap on the surface of a structure or material to determine its solidity. Similarly, percussion or tapping techniques are used by doctors in clinical examinations to determine density or cavity and assess certain conditions of the thorax and abdomen [11]. They can also be used for assessing conditions in other parts of the body. Percussion sound transmitted through bones, listened through the chest using a stethoscope, was reported to be used to detect osteoporosis [12]. A close correlation between bone resonant frequencies and the BMD was confirmed recently [13]. The lowest resonant frequency of tibia and other physiological information were mapped onto the fracture risk assessment tool (FRAX) algorithm to give a diagnosis of osteoporosis [14]. More recently, the authors proposed a machine learning method to differentiate vibro-acoustic signals and detect osteoporosis [15]. 

Therefore, the aim of the project is to identify and use these parameters from the bone’s impulse response (IR) to classify individuals which likely have OP from the general population. 

This aim will be supported with the following hypotheses: the parameters can be reliably generated and detected using common clinical equipment, and the machine learning algorithm can learn the trends of the populations and classify them correctly. 

**Figure 1:**
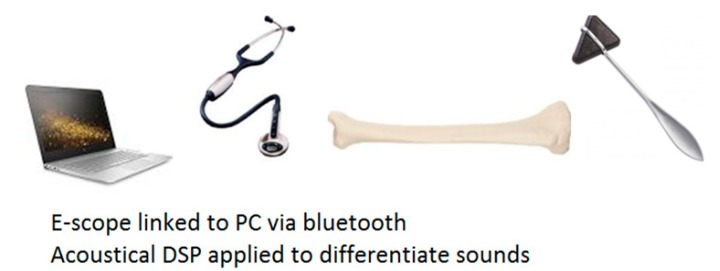
An illustration of a practical method for an osteoporosis screening testing.

This paper details a new vibro-acoustic method with the potential to diagnose osteoporosis from the impulse responses of the tibia in vivo and presents an additional machine learning approach and results. The method is illustrated in Figure 1. A clinician taps on a patient’s proximal tibia bone with a Taylor reflex hammer and an electronic stethoscope picks up the induced sound at the midpoint and/or the distal end of the tibia. The signal is transmitted via a Bluetooth datalink to a computer for further signal processing and pattern recognition, leading eventually to a diagnostic decision. By utilizing common clinical devices and apparatus, the method has considerable potential to be used by primary care providers as a screening test for whole populations, thus enabling early detection of osteoporosis.

The diagnostic decision-making mechanism of the proposed method is based on statistical machine learning from a large number of recordings. The machine learning algorithm maps the individual impulse responses to a continuous output, which is then split into two classes: healthy (OK) and osteoporotic (OP). This is based on the doctors’ diagnoses of the patients, taking into account DEXA t-scores and other physiological parameters and aspects. The development of such a signal pattern recognition method is effectively a hypothesis testing process. This is based on the assumption that information about bone mineral density, porosity and other determining factors of osteoporosis is contained in the impulse responses of the tibia. It follows that this information is adequate to make it possible to separate osteoporotic cases when data is projected onto a suitable high dimensional space. Heuristically, one can find many reasons to postulate that osteoporosis might be detected from tibia impulse responses in vivo and machine learning is a possible solution to diagnostic decision-making.

The lowest resonant frequency is closely related to the bending stiffness of the bone and, therefore, the quality of the bone [16,17]. This is in line with what is found in a simple rod model:

(1) f0= km

where k (N/m) is the stiffness of the rod, m (kg) is the mass and $f_{0}$ (Hz) is the fundamental frequency. Reduced stiffness results in a lowered fundamental frequency. While having osteoporosis does result in reduced stiffness of the bone, it also removes mass from the bone [18]. Such a decrease in the mass will counteract the reduction of the lowest resonant frequency. It is also observed that the pores formed due to bone losses are filled with fat and other body substances, and, therefore, the mass reduction is not as significant as just forming cavities. Much of the research indicated that lowered resonant frequencies and shifting in modal frequencies’ distribution are associated with osteoporosis, although the strict proportional relation between the resonant frequency and the square root of stiffness does not hold, due to the fact that mass is a yet another dependent variable [19,20,21,22,23].

Clear correlation relationships between resonant frequencies of long bones and whole body BMDs are evident, which has recently been confirmed [13]. There are other successful paradigms of using precession techniques and so acquired impulse responses in vivo to detect osteoporosis, e.g., [12,24]. However, a more thorough meta-analysis would be needed to investigate the effects of different physical parameters on the bone’s frequency response, especially the bone length, bending stiffness etc.

Bone has a complex anisotropic internal structure, which is made of two main layers: a surface layer of calcium and an internal network formed by trabeculae: a porous mass of rods and branches. The trabeculae are arranged in cross-connected plates along the bone to give strength in typical load directions [25]. Modes of vibration found in a long bone, therefore, depend on their axis and type, each with a different stiffness. The complexity and diversity in the bony structures of individuals make accurate mathematical modeling and analytical solutions of the governing equations extremely difficult. Machine learning methods for this type of complex problems are a sensible choice if a large number of examples and reliable “teacher” values can be made available.

Impulse responses acquired in vivo using percussion techniques have artifacts: The Taylor reflex hammer has a semi-rigid rubber head and the soft-tissue layers introduce a damping effect. Therefore, the impulse responses include convolved components of these damping/soft layers, which vary considerably in a population. Feature extraction methods that can de-convolve complex signals might be beneficial. Machine learning may be expected to learn from a large number of examples to disregard these components found in signals.

## 2. Materials and Methods

### 2.1. Impact Source and Vibro-Acoustic Transducer

A standard method to study vibration phenomena and the characteristics of a mechanical structure is to measure the impulse responses using an impact hammer to generate an impulsive stimulus and accelerometers as transducers at various excitations and receiving positions to pick up vibro-acoustic signals. Previous studies of resonant frequencies of the long bones, both in vivo and in vitro, used accelerometers exclusively to pick up signals. For in vitro studies, impact hammers or the like were used; alternative impact sources were used in vivo, e.g., a 20 g glass ball suspended from a 20 cm cotton string [13]. However, standard accelerometers are not suitable for clinical adoption, owing to their unfamiliarity to healthcare professionals and difficulty in mounting. 

**Figure 2:**
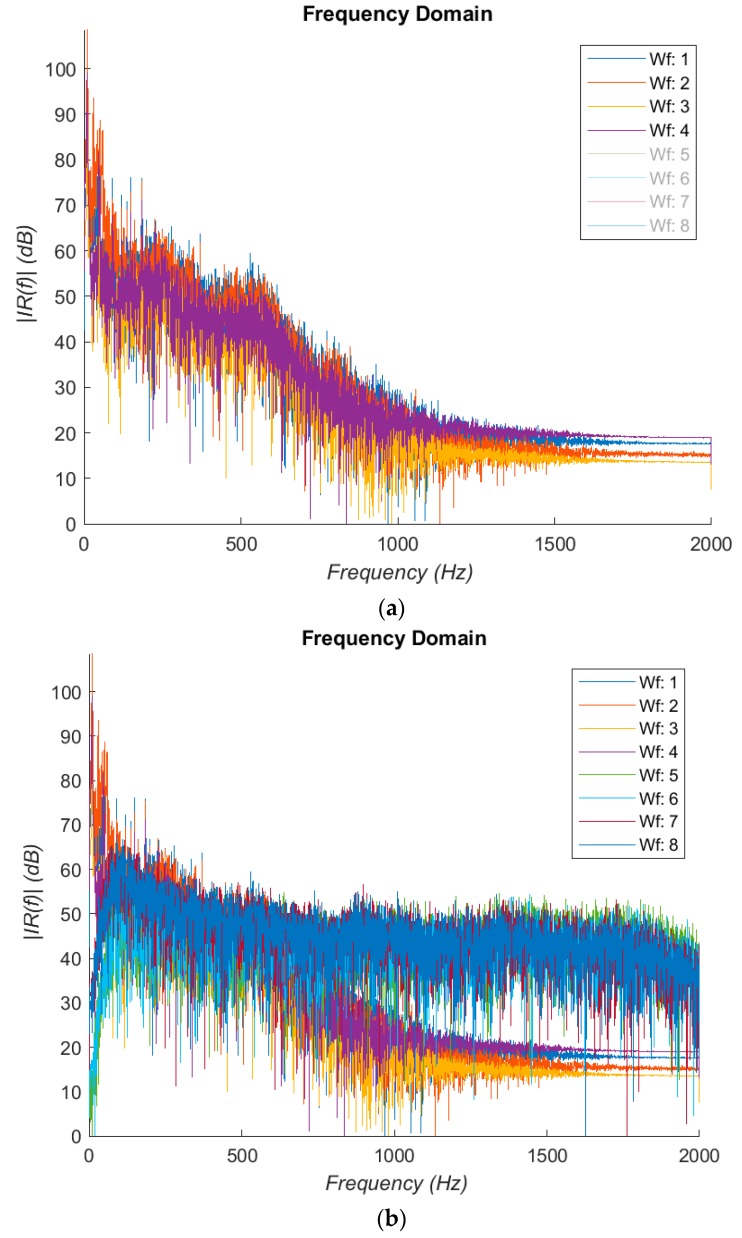
(**a**) Frequency response of stethoscope from a noise signal. (**b**) Overplayed stethoscope and accelerometer signals, Tracks 1–4: stethoscope; Tracks 5–8: accelerometer.

To ease the vibro-acoustic data acquisition in a primary care clinical setting, the use of reflex hammer as an electronic-stethoscope was first proposed by the authors [15]. To exploit the full bandwidth of the electronic stethoscope, the “extended range” filter mode is selected in the StethAssist software on exporting the audio files. To explore the frequency response of the stethoscope, an experiment was set up to understand its response to vibration signals from a broadband shaker (Ling Dynamic Systems V101/2—PA 25E [26]) driven by a white noise (Siglent SDG 1050 [27]) alongside an accelerometer (Brüel and Kjær Type 4507-B-004 [28]), with the accelerometer as the reference. Figure 2a,b displays the results. It was noted that there are a substantial resonances in the 10–40 Hz region; the frequency response is generally flat till 600 Hz.

This is followed by a −24 dB/Oct rolling off, extending to 1.2 kHz. Spectral components above 1 kHz are heavily attenuated in impulse responses measured in vivo due to soft tissues and can hardly be usefully detected even with accelerometers. This suggests that the percussion method examines only resonant frequencies in a relatively low audible frequency range, and the electronic stethoscope seems adequate for the purpose of the current study. If preferred, the frequency response can be equalized.

The recorded stethoscope signal is the convolution of the impulse responses of the bone, the soft tissue as well as all the devices in the signal chain:

(2) $$y\left(t\right)=B\left(t\right)⨂S_{ti}\left(t\right)⨂H\left(t\right)⨂S_{th}\left(t\right)$$

where y is the recorded signal, B is the bone response, $S_{ti}$ is the soft tissue response, H is the Taylor hammer and $S_{th}$ is the stethoscope response. (Modelling soft-tissue as a linear sub-system by an impulse response may not be accurate, due to non-linearity). To isolate the sound of the bone and soft tissue, the response of the signal chain has to be removed. This can be done by taking an impulse response of the devices in the chain, and filtering their responses in the frequency domain.

(3) $$Y\left(F\right)=B\left(F\right)\times S_{ti}\left(F\right)\times H\left(F\right)\times S_{th}\left(F\right)$$

(4) $$B\left(F\right)\times S_{ti}\left(F\right)=\frac{Y\left(F\right)}{H\left(F\right)\times S_{th}\left(F\right)}$$

This can be simplified by grouping the signals into:

(5) $$L\left(F\right)=\frac{Y\left(F\right)}{C\left(F\right)}$$

where L represents the whole limb (bone and soft tissue) and C is the signal chain (the hammer and stethoscope). Straightforward division in the frequency domain is not preferred due to its high sensitivities to noise found in the denominator. A linear phase (symmetrical) finite impulse response filter with 63 taps was found to be adequate to offer a reasonable equalisation. 

### 2.2. Dataset

Recordings of 110 patients were made by Rakoczy in 2016 and 2018 following the method described in Figure 1. A summary of the patient statistics is given in Table 1. These recordings were taken from the midpoint of the tibia and are processed by a MATLAB script to extract the individual impulse responses (IR). The IR are transformed into the time-frequency domain by Fast Fourier Transform (FFT) and expressed as Mel Frequency Cepstrum Coefficients (MFCCs), the parameters of which are described in Table 2.

### 2.3. Signal Pre-Processing

**Figure 3:**
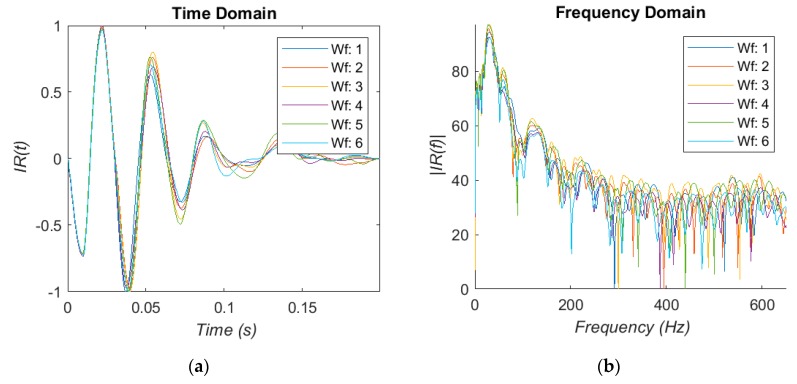
(**a**) Individual IR extracted and sorted. (**b**) The resulting FFT of IR.

The recordings of the IR are too large and noisy to be used as-is. Therefore pre-processing is needed to extract and collect the individual IR for further analysis. This is done with a two stage process using the waveform gradient and amplitude envelope to find the IR from the recording and discard the rest. A simple error checking stage then removes low frequency rumble and short impulses from the selections. This can be broken by loud impulses or clicks similar to the IR of the bone, but is able to correctly extract at least 80%of the total IR from a recording. The responses are collected and displayed in Figure 3a.

The signals are transformed into the frequency domain using the FFTW algorithm to compare the recordings with the literature findings [29]. Owing to the very short sample length of the impulses and the low sample rate, the window length of the algorithm is made much larger by zero padding to increase the frequency resolution. The results are shown in Figure 3b. There is a peak in the very low frequency region, which we assume to be the resonance of the stethoscope as described previously. A region of peaks is found at 75–110 Hz, followed by a cluster of much more damped peaks in the 200–250 Hz region. These regions are consistent with the literature [13,14,15,16,17,18,19,20,22,23], but there is not a clear change in the peak position to suggest a strong correlation. The decrease in t-score did not result in a reliable decrease in the resonant frequency. Therefore, using the fundamental frequency alone was deemed insufficient to be used for this study.

**Figure 4:**
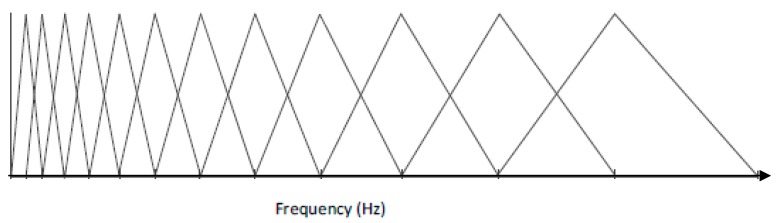
Mel Filterbank shapes.

**Figure 5:**
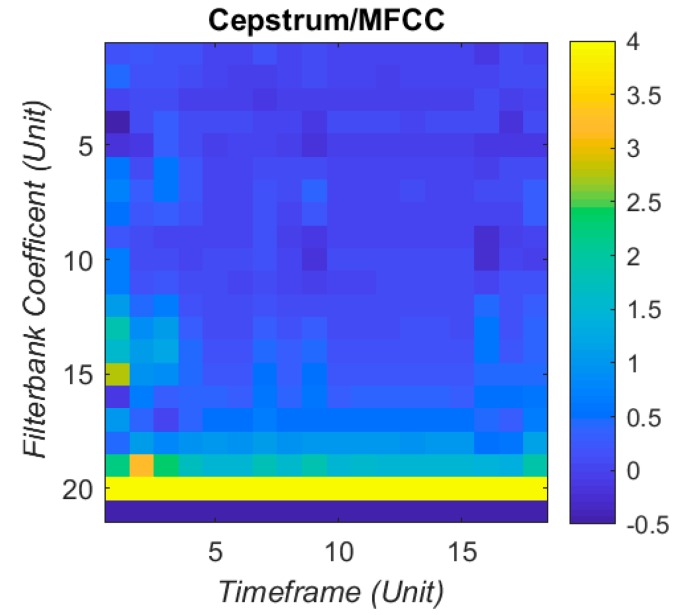
Cepstrum coefficient sum of IR. (Colour bar is clipped to increase resolution).

To reduce the data points and mitigate the complexity of machine learning, time-frequency domain representations, namely MFCCs, are used to capture the features and resonance patterns. The MFCCs have found popular use in speech recognition and music classification, but are used for this project for both the cepstrum de-convolution properties and the Mel Frequency filterbanks. The Mel scale is the subjective measurement of pitch from frequency as studied in psychoacoustics. The coefficients in MFCC represent the energy in each filterbank, and for this project 21 coefficients are used. The filterbank shape and distribution is described in Figure 4. Each set of MFCCs has 21 coefficients, taken over 18 time windows. This produces 378 coefficients arranged as a column vector to input into the artificial neural network (ANN). The visual sum of all the IR together is displayed in Figure 5, with a clipped colour bar to improve reduce the influence of peaks in the MFCC.

Filtering can be applied at different stages of the extraction process, but this is not necessary because the ANN will be able to ignore any common low frequency effect in the recording. This is also made redundant from the de-convolution from transforming into the cepstrum domain. Any consistently low value coefficient in the MFCC can be considered irrelevant and removed from the input to the ANN.

### 2.4. Machine Learning Methods

Machine learning (ML) can be thought of as an error reduction algorithm which maps input data in a non-linear fashion to desired or statistically significant outputs. There are several ML algorithms such as decision trees and support vector machines and ANN. For this project, the ANN was chosen for its fast learning capabilities and convenience.

ANNs are made from a large number of interconnected neuron models which allow a learning capability [30]. The neuron features a weighted input sum from the other neurons, which is then passed to an activation function. The activation function then shapes the summed and weighted inputs into a single output, either as binary logic via a threshold or as a sigmoid function for a continuous output bounded in the interval (0, 1). A continuous sigmoid function is used in this study.

(6) $$a_{i}=\;\frac{1}{1+\;e^{-u_{i}}}$$

where $a_{i}$ is the output of the ith neuron, and $u_{i}$ is the weighted sum of the inputs into that neuron.

The input layer contains single neurons for each data point with only one input. The hidden layers reduce the data and manipulate the weights to find the optimal solution. The output layer then takes the outputs of the last hidden layer and sums them for a final result. The network was built with 378 neurons for the input layer, 120 for one hidden layer and 1 output neuron. For larger datasets, this is modified to include a second hidden layer of 40 neurons.

The ANN starts with random weights and the MFCC is passed through the ANN. The output of the network is compared with the doctor’s diagnosis (“teacher value”) and the error between them is calculated. The teacher values are: 0 for osteoporotic (OP) patients; 1 for healthy (OK) subjects. The teacher values are informed by senior doctors’ diagnoses of the patient in question, which are generally in line with the aforementioned WHO guidelines i.e., t-scores below −2.5 as osteoporotic. But some other conditions and aspects are included in the diagnosis such as lifestyle and family history, which becomes part of the teacher value. The aim of training is to minimise the total square error *E* as defined in Equation (7) over all training examples:

(7) $$E=\frac{1}{2}\;\sum_{m=1}^{M}{\left[o^{\left(m\right)}-t^{\left(m\right)}\right]}^{2}$$

where o is the ANN output t is teacher value *m* is example number.

This error is then used in the back-propagation algorithm which updates the weights for each neuron [30]. This process means that the most important or distinctive data points in each layer are weighted more heavily in the sums and decisions than other points, reducing the data required to represent the information.

### 2.5. Classification and Voting Stage

**Figure 6:**
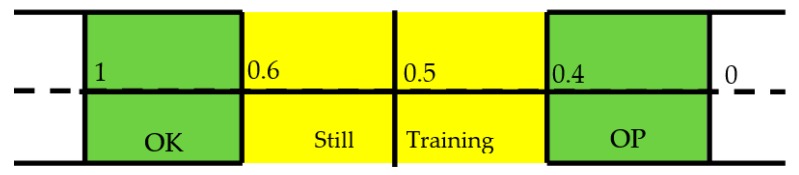
Error threshold diagram. Green indicates the catchment areas for classifying OK or OP. Yellow indicates excluded ambiguous results.

The ANN is designed with a continuous output for the following reason: while the aim of using machine learning algorithms is to separate the OP cases from the healthy through a binary classifier, the third WHO classification of osteopenia means there is a need for a greater resolution of results. Therefore, the output of the ANN is passed through a post-hoc stage which classifies the output on the error within a threshold of the teacher value. This is best explained in Figure 6:

The IR is deemed as being OK or OP if its error from the teacher value is within 0.4. If the error is substantial then it is classified as a false positive or negative. A score in the middle of the scale of 0.5 is ambiguous as to if a decision has been made, and so is removed from the decisions on the overall patient. The results from this stage for each patient’s IR are collected and the diagnosis of the patient is made from the majority of decisions made. This voting stage is key to deciding the overall diagnosis of the patient, as it removes the emphasis on individual IR decisions.

### 2.6. Training and Validation

The dataset is split between training dataset and validation dataset. The former teaches the ANN on examples which have teacher values in order to reduce the error. The latter tests the network on examples it has not “seen,” proving the ANN is able to be used on new data. For the initial pilot study with 12 patients, the training set includes 48 impulse responses and the validation set contains 46.

The algorithm is being judged on how many OP cases it can accurately detect while having a reasonable false positive rate. For a screening test, the emphasis is to find as many cases as possible from a population, and so false negatives have to be reduced, at the expense of false positives rising. Therefore, three main parameters are to be calculated: true positive rate (TPR), false positive rate (FPR), and accuracy (ACC). The first parameter is described as the sensitivity (SEN) in medical terminology, defined as:

(8) $$TPR=SEN=\frac{TP}{P}$$

where TP is the number of true positives, and P is the number of actual positive cases.

The second term, false positive rate, is calculated as:

(9) $$FPR=\frac{FP}{N}$$

where FP is the number of false positives and N is the total number of negatives cases in the population. However this statistic is better understood as the ratio of true negatives to real negatives, described in medical terms as the specificity (SPC), which is defined thus:

(10) $$TNR=SPC=\frac{TN}{N}=1-FPR$$

where TN is the number of true negatives, and N is the total number of real negative cases.

Finally, accuracy describes the balance between true positives and true negatives. This can be a very useful metric for when the number of positive and negative cases are not equal. This is expressed as:

(11) $$ACC=\frac{TP+TN}{P+N}$$

where the definitions follow as before.

Lastly, a ratio between the FPR and TPR was made to judge the performance of the algorithm and be used as a way of recording the maximum sensitivity and minimum FPR together.

(12) $$r=\;\frac{FPR}{TPR}$$

r is calculated both on an individual IR level and on a patient level. Therefore, it will separately judge the performance of the algorithm on individual datapoints and entire cases.

Criteria were set to judge the algorithm’s performance, and when they are reached the current state of the ANN is saved (i.e., weights, bias and training time). These would apply for every run of the algorithm training. The three criteria are:

- When the ratio between false positives over true positives reaches a minimum (initiated when it falls below 0.7).
- When maximum accuracy is reached.
- When the training error dropped below a threshold.

The first criterion is to establish that the method can be used as a screening method to detect OP cases accurately. The second is to investigate if the method is able to differentiate the healthy subjects well enough and reduce the false negatives and positives. The third is the stop criterion of the algorithm, and will indicate when overfitting is beginning to occur.

The patients are decided by the thresholds described in Figure 6. These were set to avoid ambiguity in interpreting outputs around 0.5, and to imply that there is space for a third category in the future. However, for this investigation it was useful to see if the above criteria are affected by a change in the thresholds that define the OP and OK population. Therefore, the training was repeated by offsetting the thresholds to range from 0.2 and 0.6 to 0.5 in 0.005 increments.

## 3. Results

**Figure 7:**
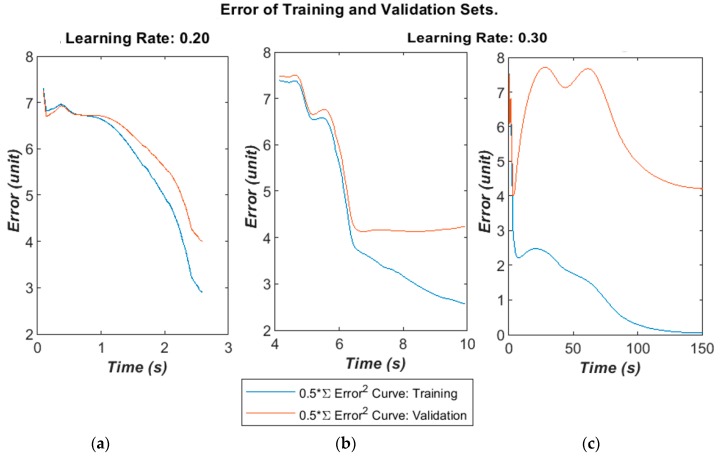
(**a**) Training and validation dataset error falling together. (**b**) Errors begin to diverge. (**c**) Training error starts to overfit, while validation error converges to an error energy of 4.

The algorithm’ error over time is given below in Figure 7a–c for different learning rates and selected runs. The time was calculated in the MATLAB script running on a Panasonic CF-52 laptop (2 CPU cores@2.4 GHz with 4 GB RAM). The error was calculated as the half error energy defined in Equation (7).

The training and validation follow a similar curve in the very early stages of the training, but diverge after a moderate time (400–1000 epochs) into the run. On some runs, the validation error will greatly increase as the algorithm begins to fall into overfitting.

**Figure 8:**
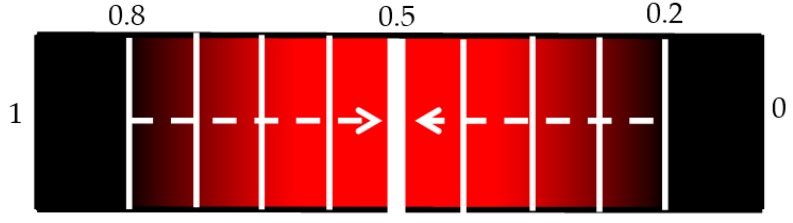
Changing threshold level. Starting at the smallest thresholds (0.2 and 0.8), the thresholds are increased until they meet at the middle.

It is useful to show the relationship between SEN and 1-SPC using a continuous receiver operating characteristic (ROC) graph to illustrate the algorithm’s detection performance. Because of the limited computing power of the laptop only a limited number of points could be made which meant a quasi-ROC graph had to be made using discrete scatter points. While this is not ideal, it is more convenient for using the limited power available. Also, since the performance of a single algorithym is taken, there is no need to calculate the area under the graph, so discrete quasi-ROC is more appropriate. Each 0.005 change in the threshold is indicated on a colour bar like in Figure 8. The darker shades indicate “smaller” thresholds (0.2 and 0.8 to detect OP/OK subjects) while the red colour is closer to a complete binary decision (0.5).

**Figure 9:**
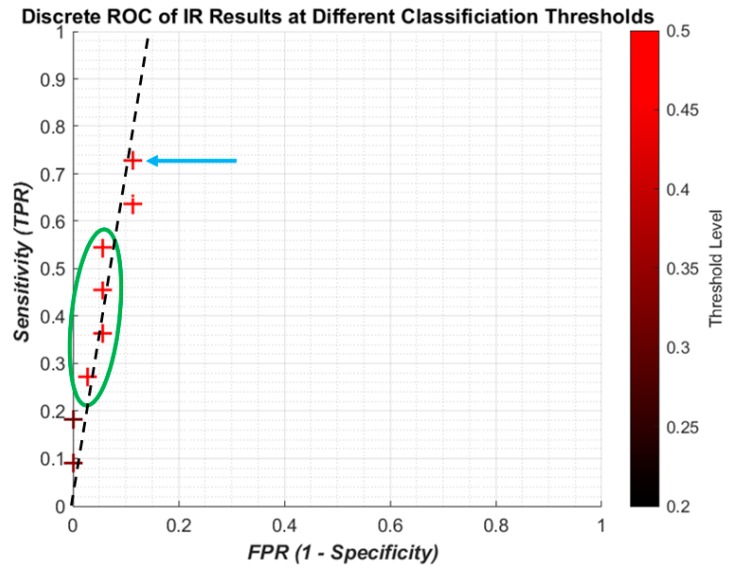
Discrete receiver operating characteristic ROC graph showing the IR sensitivity against 1—Specificity. Blue arrow is the best collective result; the green circle indicates foldback in the results, with higher thresholds not giving better results linearly; dashed black line is manually applied line of best fit.

The discrete ROC plot for individual IR is shown in Figure 9. The small dataset and random initial weights means there are occasionally repeated results, so at some point only the result from the higher threshold, the lighter colour, is shown. 

The lower thresholds (dark crosses) result in the poor sensitivity, and with higher thresholds (redder crosses) the sensitivity increases with a small decrease in specificity. However it can be seen that the best result (blue arrow) is not the highest threshold, and there is some foldback with regard to the results (green circle). Since the change in threshold does not result in a linear improvement with sensitivity/specificity, there is no attempt to apply regression curve techniques between points. However, it is clear that all the results are highly specific, being on the far left of the plot. A likely line of best fit is shown as the dashed black line, showing high specificity with improving sensitivity.

**Figure 10:**
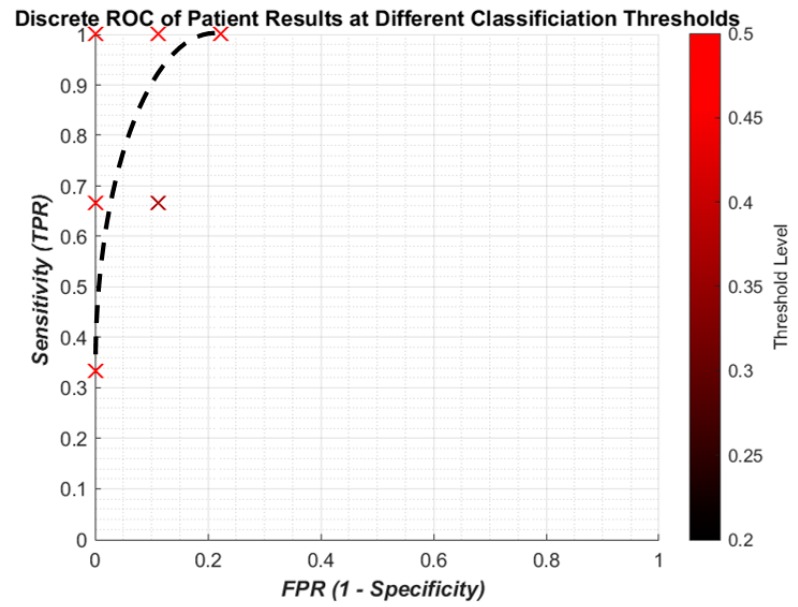
Discrete ROC of patient results showing clustering in North West quadrant, implying a high sensitivity with a high specificity. Dashed black line are suggested curves, not from regression.

Figure 10 gives the results for the sensitivity and specificity of detecting OP/OK patients by the algorithm. The results are much more promising, with a “perfect” result of 100% sensitivity to 0% FPR. With a small dataset of 12 patients, this is more likely to be possible and, therefore, should not be taken to mean that the algorithm has achieved the goal. Indeed while the result does indicate that the ANN is able to find the population trend between OP and OK subjects, the patient result is due to the post hoc rules (Section 2.5) weighted against incorrect IR predictions. Therefore, a larger dataset should be tested on to see if similar results are indicated. Overall, in summary, lower thresholds tend to give worse sensitivities for both IR and patients, while higher thresholds closest to 0.5 improve sensitivity but this is not consistent.

**Figure 11:**
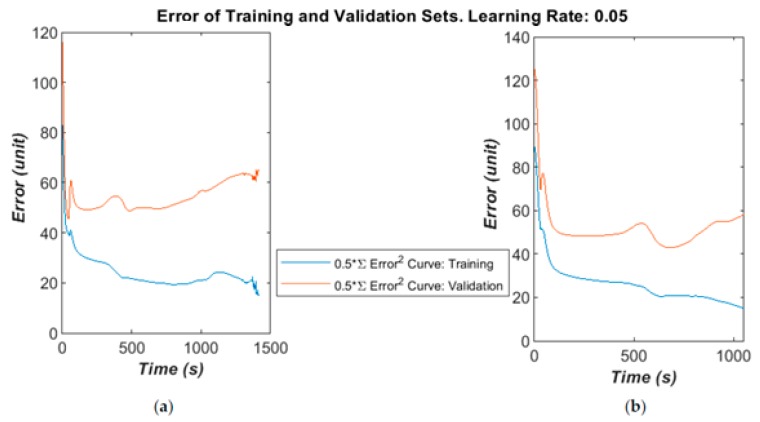
(**a**) Extended training and validation dataset’s error diverging. (**b**) Validation error increases in the late stage.

A larger dataset was available to test the ANN and develop the method, containing 110 patients: 34 OP and the rest healthy. The total number of impulse responses used was 660:330 for training and 330 for validation. A block updating technique was adapted, in which the weights were updated and error calculated after presenting the whole set of training data. The algorithm found a reasonably good minimum fairly quickly in validation testing then started to over-fit, as shown in Figure 11a,b. This indicates that the dataset could contain some noise or irrelevant information, and might benefit from some better signal conditioning or a more robust feature space. However, it is worth noting that a minimum error below 50 is generally guaranteed, which is calculated from validation testing over 330 examples by Equation (7). This gives a maximum error rate of 30% or a minimum correct recognition rate of 70%. These are in line with the results from the small-scale study with 12 patients.

A summary of a selection of results are given in Table 3:

## 4. Discussion

The pilot study comprised of a limited training and validation dataset, but there are some promising results to indicate that the machine learning approach using the MFC is the right approach. Firstly, the training error converges in less than 5000 epochs, while the validation error in the same span of time varies but converges in the same way. While not a perfect cross-validation, it does indicate that the ANN can find the features in the dataset which can be used to separate the healthy subjects from the osteoporotic patients. The 12 patient training and validation datasets were linked by being from the same patients, which was a compromise with the small number of patients available. Therefore, the statistical power is poor and trends not very clear. However, this does allow for more informed individual analysis of ANN results which could not be realistically done with a much larger dataset. This can be done to see which IR or patients are always successful or give the algorithm difficulty.

From the ROC graphs, it is clear that the sensitivity to individual IR is limited but avoids a high number of false positives. However, this low level does not affect the sensitivity of the patient predictions, meaning that the algorithm does not require every impulse from a patient’s recording to indicate osteoporosis. Instead this allows for some minor misclassification to occur without jeopardizing the diagnosis of the patient. The change in threshold shows that capturing more of the impulses that are between the two classification catchment areas (0.4–0.6) improves the sensitivity without a large increase in FPR. However a more detailed investigation would be needed to see if the threshold change consistently gave better predictions.

The results indicate that the algorithm was able to have 100% patient sensitivity without any false positives. It should be noted that a limitation of the results is with such a small dataset, the patient sensitivity result is not indicative of performance with a larger dataset. However, it does show promise, and with the larger dataset of patients and revision to the ANN architecture this could show promising results.

For a screening test, the results here indicate that this method could be suitable for use once the development has reached the right point. Firstly the test is non-invasive which is patient friendly. The clinician carrying out the method needs only a computer running the client-side software, and the hammer with the electronic stethoscope, while the training can be done centrally. The test and results seem not to be sensitive to the variation in impacts, but this ought to be confirmed in experimental trials.

Overall, there is potential in this method and many areas which can be taken further. The priority in future work is to improve the size of the dataset to continue to decrease the validation error as the number of examples increase. Cross-validation will be carried out more formally by using a designed test set to improve the generality of the algorithm on an independent dataset. This will improve the machine learning foundation of the method, while other areas will be optimising the data processing and experimental technique.

The results so far indicate that the ANN is able to identify and weight the trends of the OP and OK populations differently, which is a key step in the right direction. To the author’s best knowledge, there has not been a similar result from another device or method which is able to diagnose the OP from the OK while using clinical tools. Indeed, this is the key strength of this method and study in particular.

However, this must be balanced with some limitations in this paper’s results. Firstly, the pilot study is still rather small at 12 patients with just under 100 IR to test and validate. The algorithm was able to overfit to the data because of its small size, and so results should be taken with caution. The discrete ROC graphs are not the ideal way of presenting the performance of the algorithm, since it is more usual to have a continuous curve. However, with refinement to the algorithm and better understanding of what parameters can be changed, this can be improved.

## 5. Conclusions

A proposed method for the screening of osteoporosis has been developed in this paper. Using two pieces of common medical apparatus—the reflex hammer and the electronic-stethoscope, along with a computer script based on an ANN—the method has been developed to be intuitive and easy to implement for GPs and nurses in healthcare systems. While machine learning methods are seen as a ‘blackbox’ approach to complex problems, the method proposed in this paper is grounded on the relevant physical parameters of bone such as stiffness, mass and porosity. As a proof of concept pilot study, the paper only used a limited dataset of 12 patients for the initial designs of the ANN. Even so, a sensitivity of over 60% was achieved for both patients and IR. Results from the larger dataset are in line with the above findings with respect to error energy in proportion to number of samples. With further work on the signal-conditioning stage, feature extraction and selection algorithms, as well as ANN architecture, this can be improved, and sensitivities and specificities quantified. With further refinement to the algorithm and data representation, the results and application of the method can become clinically useful.

## Figures and Tables

**Table 1:** Population Statistics.

Parameter	Mean [Min, Max]
Gender	M: 19; W: 90
t-score	−1.33 [−3.5, 0.6]
No. Osteoporosis (OP) Cases	34
No. Healthy (OK) Cases	76

**Table 2:** Dataset parameters.

Parameter	Size	Unit
Impulse Responses (IR) Sample Length	800	samples
Fast Fourier Transform (FFT) Window Length	8192	points
FFT Output Spectra Size	100	points
Mel Frequency Cepstrum Coefficients (MFCC) Window Length	0.03	s
MFCC Window Overlap	0.02	s
MFCC Total	378	points

**Table 3:** Summary of Parameters for Selected Thresholds.

Threshold	$r_{IR}$		$r_{PAT}$	
(0.050 increments for table)	TRP	FPR	TRP	FPR
0.200	0.5455	0.0571	0.6667	0
0.250	0.4545	0.0571	0.6667	0
0.300	0.0909	0	0.3333	0
0.350	0.0909	0	0.3333	0
0.400	0.4545	0.0571	1.0000	0.1111
0.450	0.4545	0.0571	0.3333	0
0.500	0.7273	0.1143	1.0000	0.1111

## References

[1] International Osteoporosis Foundation https://www.iofbonehealth.org/what-is-osteoporosis.

[2] Kastner M., Perrier L., Munce S.E.P., Adhihetty C.C., Lau A., Hamid J., Treister V., Chan J., Lai Y., Straus S.E. (2018). Complex interventions can increase osteoporosis investigations and treatment: A systematic review and meta-analysis. Osteoporos. Int..

[3] Ström O., Borgström F., Kanis J., Compston A., Cooper J., McCloskey C., Jönsson E. (2001). Osteoporosis: Burden, health care provision and opportunities in the EU. Arch. Osteoporos..

[4] Kanis J.A., Johnell O., Oden A., Jonsson B., De Laet C., Dawson A. (2000). Prediction of Fracture From Low Bone Mineral Density Measurements Overestimates Risk. Bone.

[5] Kanis J.A., WHO Study Group (1994). Assessment of Fracture Risk and its Application to Screening for Postmenopausal Osteoporosis: Synopsis of a WHO Report. Osteoporos. Int..

[6] Bouxsein M.L. (2003). Mechanisms of Osteoporosis Therapy: A Bone Strength Perspective. Clin. Cornerstone.

[7] Felsenberg D., Boons S. (2005). The bone quality framework: Determinants of bone strength and their interrelationships, and implications for osteoporosis management. Clin. Ther..

[8] Pulse, Osteoporosis QOF to Be Hit by Long DXA Waits. https://search.proquest.com/docview/993338099.

[9] Pulse, OSTEOPOROSIS: Expert Criticises New Osteoporosis QOF Targets. https://search.proquest.com/docview/902814749.

[10] Lenus, Access to Diagnostics: A Key Enabler for a Primary Care Led Health Service. https://www.lenus.ie/handle/10147/292726.

[11] McGee S.R. (1995). Percussion and physical diagnosis: Separating myth from science. Dis. Month.

[12] Stagnaro M.N., Stagnaro S. (1991). Diagnosi clinica percoce dell’osteoporosi con la percussione ascolta. Clin. Ter..

[13] Razaghi H., Saatchi R., Huggins T., Bishop N., Burke D., Offiah A.C. Correlation analysis of bone vibration frequency and bone mineral density in children. Proceedings of the IEEE 2014 9th International Symposium on Communication Systems, Networks & Digital Sign.

[14] Tejaswini E., Vaishnavi P., Sunitha R. Detection and Prediction of Osteoporosis using Impulse response technique and Artificial Neural Network. Proceedings of the IEEE 2016 International Conference on Advances in Computing, Communications and Informatics (ICACCI).

[15] Scanlan J., Li F.F., Umnova O., Rakoczy G., Lövey N. (2018). Machine learning and DSP Algorithms for Screening of Possible Osteoporosis Using Electronic Stethoscopes. Proceedings of the 3rd International Conference on Biomedical Imaging, Signal Processing (ICBSP 2018).

[16] Jurist J.M. (1970). In vivo determination of the elastic response of bone. I. Method of ulnar resonant frequency determination. Phys. Med. Biol..

[17] Jurist J.M. (1970). In vivo determination of the elastic response of bone. II. Ulnar resonant frequency in osteoporotic, diabetic and normal subjects. Phys. Med. Biol..

[18] Doherty W.P., Bovill E.G., Wilson E.L. (1974). Evaluation of the Use of Resonant Frequencies to Characterize the Physical Properties of Human Long Bones. J. Biomech..

[19] Cornelissen P., Cornelissen M., Van Der Perre G., Christensen A.B., Ammitzboll F., Dyrbye C. (1986). Assessment of Tibial Stiffness by Vibration Testing in situ—II. Identification Influence of soft tissues, joints and fibula. J. Biomech..

[20] Christensen A.B., Ammitzboll F., Dyrbye C., Cornelissen M., Cornelissen P., Van Der Perre G. (1987). Assessment of Tibial Stiffness by Vibration Testing in situ—III. Sensitivity of Different Modes and Interpretation of Vibration Measurments. J. Biomech..

[21] Jurist J.M. (1973). Letter: Difficulties with measurement of ulnar resonant frequency. Phys. Med. Biol..

[22] Holi M.S., Radhakrishnan S. In vivo Assessment of Osteoporosis in Women by Impulse Response Technique. Proceedings of the TENCON 2003. Conference on Convergent Technologies for Asia-Pacific Region.

[23] Bediz B., Özgüven N.H., Korkusez F. (2010). Vibration measurements predict the mechanical properties of human tibia. Clin. Biomech..

[24] Moore M.B. (2009). The use of a tuning fork and stethoscope to identify fractures. J. Athl. Train..

[25] Rosen C.J., Langton C.M., Njeh C.F. (2004). Anatomy, physiology and disease. The Physical Measurement of Bone.

[26] Ling Dynamic Systems. http://www.crtech.co.uk/pages/environmental-testing/v406.pdf.

[27] Siglent https://mediacdn.eu/mage/media/downloads/SDG1000_datasheet_en.pdf.

[28] Brüel & Kjær https://www.bksv.com/-/media/literature/Product-Data/bp1841.ashx.

[29] FFTW http://fftw.org/faq/section4.html#howworks.

[30] Rojas R. (1996). Neural Networks—A Systematic Introduction.

